# Postmortem proteomics to discover biomarkers for forensic PMI estimation

**DOI:** 10.1007/s00414-019-02011-6

**Published:** 2019-03-12

**Authors:** Kyoung-Min Choi, Angela Zissler, Eunjung Kim, Bianca Ehrenfellner, Eunji Cho, Se-in Lee, Peter Steinbacher, Ki Na Yun, Jong Hwan Shin, Jin Young Kim, Walter Stoiber, Heesun Chung, Fabio Carlo Monticelli, Jae-Young Kim, Stefan Pittner

**Affiliations:** 1 Graduate School of Analytical Science and Technology (GRAST), Chungnam National University, Daejeon, South Korea; 2 Dept. of Biosciences, University of Salzburg, Salzburg, Austria; 3 Dept. of Integrated Mathematical Oncology, H. Lee Moffitt Cancer Center & Research Institute, Tampa, FL USA; 4 Dept. of Chemistry, Sogang University, Seoul, South Korea; 5 Biomedical Omics Center, Korea Basic Science Institute, Ochang, South Korea; 6 Dept. of Forensic Medicine, University of Salzburg, Salzburg, Austria; 7 Division of Bioconvergence Analysis, Korea Basic Science Institute, Ochang, South Korea

**Keywords:** Postmortem interval (PMI), Skeletal muscle, Protein, Degradation, Proteomics

## Abstract

The assessment of postmortem degradation of skeletal muscle proteins has emerged as a novel approach to estimate the time since death in the early to mid-postmortem phase (approximately 24 h postmortem (hpm) to 120 hpm). Current protein-based methods are limited to a small number of skeletal muscle proteins, shown to undergo proteolysis after death. In this study, we investigated the usability of a target-based and unbiased system-wide protein analysis to gain further insights into systemic postmortem protein alterations and to identify additional markers for postmortem interval (PMI) delimitation. We performed proteomic profiling to globally analyze postmortem alterations of the rat and mouse skeletal muscle proteome at defined time points (0, 24, 48, 72, and 96 hpm), harnessing a mass spectrometry-based quantitative proteomics approach. Hierarchical clustering analysis for a total of 579 (rat) and 896 (mouse) quantified proteins revealed differentially expressed proteins during the investigated postmortem period. We further focused on two selected proteins (eEF1A2 and GAPDH), which were shown to consistently degrade postmortem in both rat and mouse, suggesting conserved intra- and interspecies degradation behavior, and thus preserved association with the PMI and possible transferability to humans. In turn, we validated the usefulness of these new markers by classical Western blot experiments in a rat model and in human autopsy cases. Our results demonstrate the feasibility of mass spectrometry–based analysis to discover novel protein markers for PMI estimation and show that the proteins eEF1A2 and GAPDH appear to be valuable markers for PMI estimation in humans.

**Electronic supplementary material:**

The online version of this article (10.1007/s00414-019-02011-6) contains supplementary material, which is available to authorized users.

## Introduction

A precise estimation of the time since death or the postmortem interval (PMI) is a critical issue in forensic investigations, as it can have major implications on further case investigation or even provide decisive evidence in court [1]. Especially, biomedical analysis of the corpse can provide important, dependable data. The methodic spectrum for reliable PMI delimitation on this behalf includes the examination of postmortem body cooling, supravital functions such as electrical or pharmacological excitability of body tissues or organs, and the development of rigor mortis and hypostasis [2]. In certain cases, forensic entomology [3] or analysis of morphologic changes [4] can provide a minimum PMI. These methods, however, are limited to particular postmortem phases and often cannot be sensibly applied due to specific restrictions. Hence, additional approaches are required to complement the methodic spectrum and to provide a reliable set of tools for case specific time since death estimation.

Especially, the postmortem decomposition of biomolecules has lately become of interest. In this aspect, several studies have investigated postmortem alterations of RNA [5, 6], DNA [7, 8], or proteins [9–12]. Recently, significant progress was achieved in research on postmortem degradation of skeletal muscle proteins. In standardized animal models, specific proteins were found to depict predictable degradation patterns when analyzed via SDS-PAGE and Western blotting. Postmortem changes, such as the loss of the native protein band or the occurrence of specific degradation products, were additionally shown to significantly correlate with the PMI. Among these proteins are calcineurin A, protein phosphatase 2A [13], titin, nebulin [14], desmin, troponin [15], vinculin [16], AMP-activated protein kinase, caspase 3, and glycogen synthase [17]. Furthermore, PMI-dependent desmin and cardiac troponin T degradation has been confirmed in humans [15] and was already successfully applied to obtain evidence about the succession of events in a criminal case [18]. However, to provide a broadly applicable method for time since death estimation, characterization of additional marker proteins, which undergo significant postmortem alterations, is required.

For this purpose, mass spectrometry (MS)–based proteomics approaches provide a modern technology for quantitative and qualitative assessment of proteins, enabling peptide sequencing and protein identification with high accuracy and sensitivity [19]. This technology has been shown to be a valuable tool for the characterization of protein biomarkers by the analysis of protein extracts obtained from experimental or clinical samples on a systemic level [20, 21]. Thus, this method could aid in discovering novel biomarkers for forensic time since death estimation purposes by simultaneously analyzing a high number of proteins, which is hardly achieved by conventional hypothesis-driven studies or even trial and error approaches that often require a lot of time, money, and resources. The application of MS for PMI biomarker discovery seems to be an emerging and promising field that, however, remains in its infancy yet as it is reported in only a few previous studies so far. Tavichakorntrakool et al. [22] performed two-dimensional gel electrophoresis (2DE)–based proteomics to analyze postmortem changes in human skeletal muscle proteome and suggested potential protein biomarker candidates whose abundance was altered progressively (increased or decreased) during a postmortem period (e.g., HSP27, creatine kinase). Procopio et al. [23] analyzed postmortem protein decay in bone proteome harnessing high resolution MS using pigs as a model system and found reduction of particular proteins as well as increased deamidation of biglycan with increasing PMIs.

In this study, applying high resolution MS, we aimed to profile postmortem changes in the skeletal muscle proteome in experimental animal models prepared under controlled conditions to identify potential novel protein biomarkers. As data obtained from a single species might not be easily recapitulated in human cases since species specific postmortem alteration patterns may exist, we focused on samples of two different mammalian species, rat and mouse, which were harvested during a postmortem period of 4 days (0, 24, 48, 72, and 96 hpm). Subsequently, we ran SDS-PAGE and Western blot experiments to evaluate the significance of the findings and compare the performance of the newly detected markers to others, already in use. Ultimately, we tested whether the novel markers qualify for future use in forensic routine by conducting Western blot experiments on three autopsy cases with varying PMI.

## Materials and methods

### Animal models

Twenty-five adult male Sprague Dawley rats were killed by cervical dislocation under deep isoflurane anesthesia. For Western blot analysis, immediately after death, skin, fat, and muscle fasciae of the right hind limbs of five randomly chosen rats were opened and the M. vastus lateralis was removed. Samples (0 hpm), excised from the muscle belly (approximately 5 × 5 × 5 mm, 100 mg), were snap frozen in liquid nitrogen and stored at − 80 °C until further processing. To avoid artificial desiccation and contamination effects, we used different (non-sampled) animals for each new time point. Therefore, the remaining 20 rats were immediately transferred to an environmental chamber and stored at a temperature of constant 20 °C. Humidity was monitored during the experiment and varied between 40 and 60%. At 24, 48, 72, and 96 hpm, five randomly chosen animals, respectively, were removed from the chamber and muscles were sampled as described above. For proteomic analyses, in ten randomly selected rats (*n* = 2 at each time point), a second adjacent muscle sample was excised and prepared as described. To analyze a second species on proteomic level, ten adult female ICR mice were euthanized by cervical dislocation. At each time point (0, 24, 48, 72, and 96 hpm) the M. vastus lateralis from two randomly chosen mice were harvested, frozen, and stored at − 80 °C until further processing. The remaining mice were kept in an environmental chamber at a constant temperature of 25 °C, and two animals were randomly selected for muscle dissection and sample preparation at each time point. All experiments were performed in accordance with the international ethical requirements for the use of animals in experimental research studies.

### Human cases

To evaluate the relevance of new marker proteins for time since death estimation in humans, we additionally analyzed thigh muscle samples from humans using SDS-PAGE and Western blotting. Muscle samples were gathered from routine autopsy cases at the Department of Forensic Medicine of the University of Salzburg, Austria. Only cases without prior history of muscle diseases and cooling (4 °C) times under 24 h were included. Sample H1 was taken from a 76-year-old woman who died on respiratory failure in a hospital (Table 1). Intermediate PMI is represented by a sample (H2) from a 75-year-old female who was strangled in bed and was found 2 days later (Table 1). H3 was taken from a 67-year-old man who was found dead in his apartment. He showed signs of advanced putrefaction. PMI could only be roughly estimated by non-biomedical evidence through police investigation (Table 1). Accumulated degree days (ADD), as a measure of energy affecting a system, were calculated as follows: ADD [°d] = time [d] × temperature [°C]. The presented cases remained in 4 °C cooling environment for 13.9 ± 3.0 h.

**Table 1:** Individual data of the three analyzed autopsy cases. Muscle samples were selected to represent a short (H1), intermediate (H2), and advanced PMI (H3). PMI is stated in hours postmortem (hpm) and ADD in degree days (°d)

	Age	Sex	BMI	PMI (hpm)	ADD (°d)
H1	76	F	28.7	15.8	~ 2.6
H2	75	F	22.9	40.7	~ 26.0
H3	67	M	n/a*	≥ 336	≥ 280

*Body mass index (BMI) calculation was not applicable for H3, due to advanced putrefaction and postmortem weight loss

In all cases, skin, fat, and fasciae were opened by a small incision at the lateral thigh and a biopsy sample of the vastus lateralis muscles was taken from 4 to 8 cm depth (approximately half the distance to the femur, depending on individual physique). Samples were snap frozen and stored in liquid nitrogen until further processing. Sampling of human muscle tissue for scientific purposes was approved by the ethics commission of the University of Salzburg (EK-GZ: 11/2017).

### Mass spectrometry sample preparation and LC-MS/MS analysis

The protein extracts were prepared with RIPA buffer (details of products and specific solutions are given in Online Resource 1) followed by sonication. Samples were desalted with Zeba Spin Desalting Columns and denatured in 5 M urea and 5 mM DTT (dithiothreitol) at 65 °C for 30 min and then reduced in 40 mM iodoacetamide at room temperature for 30 min. The lysates were again desalted by Zeba Spin Desalting Columns and digested with 20 μg/mL of trypsin for 2 h at 37 °C. The tryptic peptides were quantitated using Quantitative Colorimetric Peptide Assay kit. Twenty micrograms of tryptic peptides were purified by ziptip and resuspended in 20 μL of injection buffer containing 2% acetonitrile and 0.1% trifluoroacetic acid (TFA). Peptides were analyzed using a LC-MS/MS system consisting of an Easy-nLC 1200 and an Orbitrap Fusion Lumos mass spectrometer equipped with a nano-electrospray source. Protein quantitation was performed using MaxQuant Software (ver. 1.2.2.5) [24] as described previously [25]. The intensities of each protein were normalized (log2 scale) based on total intensity employing normalizer tool [26], and the normalized protein list was further processed by R and MATLAB software. For additional details including data analysis procedure refer to Online Resource 2.

### SDS-PAGE and Western blotting

Frozen muscle samples were transferred to a ceramic well and homogenized by cryogenic grinding. After adding extraction buffer (details of products and specific solutions are given in Online Resource 1), all samples were additionally homogenized by sonication and centrifuged at 1000×*g* for 10 min. The supernatant was stored at − 20 °C for further analysis. Overall protein concentration was determined by BCA assay and diluted to equal content. Samples were denatured at 90 °C for 5 min, and 10% polyacrylamide gels were used for electrophoresis. Gels were transferred to blotting membranes for immunodetection. Membranes were blocked in blocking buffer for 1 h and subsequently incubated in primary antibodies (1 h to overnight) and secondary antibodies (1 h). Between each antiserum incubation step, the membranes were rinsed and washed in washing buffer.

Antibody binding was visualized by adding chemiluminescence substrate and documented with a digital gel analysis system. Protein band intensities were measured using ImageJ software. Bands in the 0-hpm samples were considered the native form, and all alterations (disappearance of the native bands, or appearance of additional bands) were considered degradation processes. All signals < 5% the intensity of the native bands at 0 hpm were considered background and thus no band.

### Statistics

For Western blot analysis, Spearman correlation values (Spearman’s *ρ*, *p* value) were calculated for each band to assess statistically significant band changes within the investigated PMI. Kolmogorov-Smirnov tests and visual checks for normal distribution and homogeneity of variance were applied to calculated data of relative GAPDH and eEF1A2 intensities of proteomics and Western blot approaches. In case of normally distributed data, one-way analysis of variance (ANOVA) was used to test for significant differences between 0-hpm group and postmortem groups. Significance was determined by Tukey’s post hoc multiple comparisons tests. In case the data failed to satisfy the assumptions of parametric homoscedastic additive models, differences in measured variables between groups were tested by nonparametric methods. Intergroup differences were tested by the one-way rank-based analog to analysis of variance (Kruskal-Wallis test), followed by all pairwise multiple comparisons by the Bonferroni correction method. For all analyses, SPSS 23.0 software (IBM Corp) was used. *p* values ≤ 0.05 were considered statistically significant, *p* values ≤0.001 were considered highly significant.

## Results

### Postmortem alterations in rat and mouse skeletal muscle proteome

The number of protein markers currently used for forensic PMI estimation is still limited, thus, identification of additional protein markers is necessary to facilitate future application of this approach. To discover additional protein biomarkers, we analyzed postmortem alterations in skeletal muscle proteome harnessing mass spectrometry (MS)–based proteomics.

**Fig. 1:**
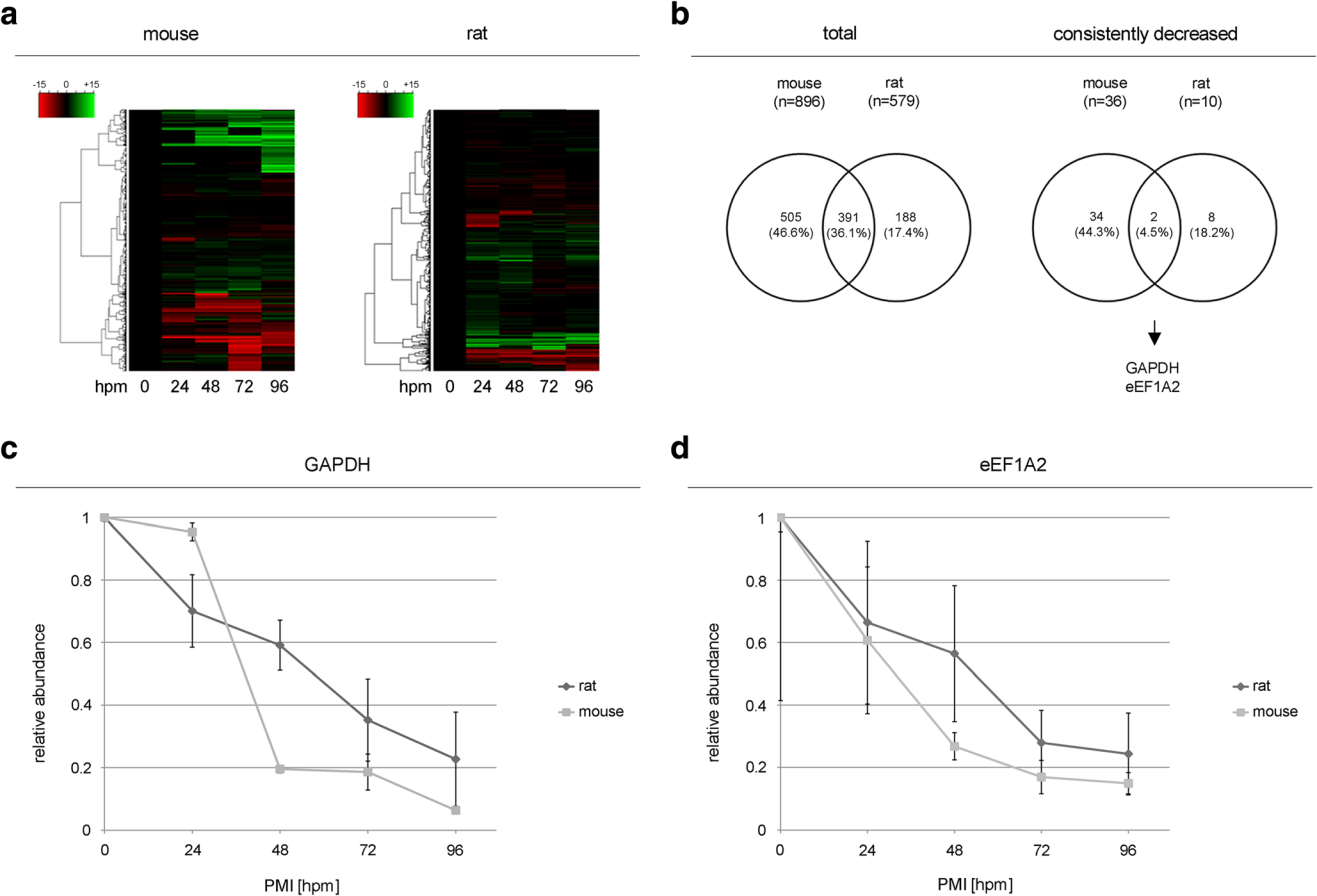
Mass spectrometry–based analysis of postmortem alterations in mouse and rat skeletal muscle proteome. **a** Hierarchical clustering and heat map of protein intensities at various hpm. Protein intensity changes from the baseline intensity were clustered using an unbiased hierarchical method with a Euclidian distance function resulting in a few different clusters. Row: protein, column: hpm. Green: increase, red: decrease. **b** Venn diagram showing the number of total (left panel) and consistently decreased (right panel) proteins. Relative abundance of GAPDH (**c**) and eEF1A2 (**d**) at various hpm. Data represented as mean ± SD

By that, we identified and quantified a total of 896 and 579 proteins from mouse and rat, respectively. An unsupervised hierarchical clustering was conducted to classify the protein intensities, resulting in several clusters in both mouse and rat cases (Fig. 1a). We focused on identifying proteins whose quantity consistently decreased (from each time point to the subsequent) as hours postmortem (hpm) increased. By that, we identified 36 and 10 consistently decreasing proteins, from mouse and rat skeletal muscle proteome, respectively. We found that GAPDH and eEF1A2 were commonly decreasing proteins in both mouse and rat (Fig. 1b). When normalizing the protein intensities to that of the 0-hpm samples in both mice, GAPDH abundance slightly decreased to 95.4% from the initial level at 24 hpm. At 48 and 72 hpm, the intensities declined by more than 80%, reaching 19.5 and 18.5% of the initial protein abundance. At 96 hpm, only 6.3% of the initial concentration was measured. In both rats, the rate of GAPDH reduction was largely consistent over time. GAPDH abundance decreased by a mean of 29.9% at 24 hpm. At 48 hpm, the concentration dropped to 59.1% from the initial value, at 72 hpm to 35.2% and reached 22.7% from the initial level at 96 hpm (Fig. 1c). The reduction pattern of eEF1A2 was similar between mouse and rat at 24 hpm decreasing by 39.2% and 33.6%, respectively. In mice, the eEF1A2 abundance declined sharply to 26.8% from the initial protein level at 48 hpm and continued to decrease to 16.9% (72 hpm) and 14.9% (96 hpm). In rats, the eEF1A2 protein decrease was continuous from 66.45% at 24 hpm to 56.5% at 48 hpm and the further decreased levels remained roughly constant at 27.9% and 24.3% at 72 hpm and 96 hpm, respectively (Fig. 1d). This decreasing trend of both proteins did not apply for statistical analysis due to the small sample size. However, to prove the applicability of those two proteins as a biomarker, we examined the postmortem alterations in another experimental setting using a larger sample size.

### Postmortem protein degradation in the rat model

**Fig. 2:**
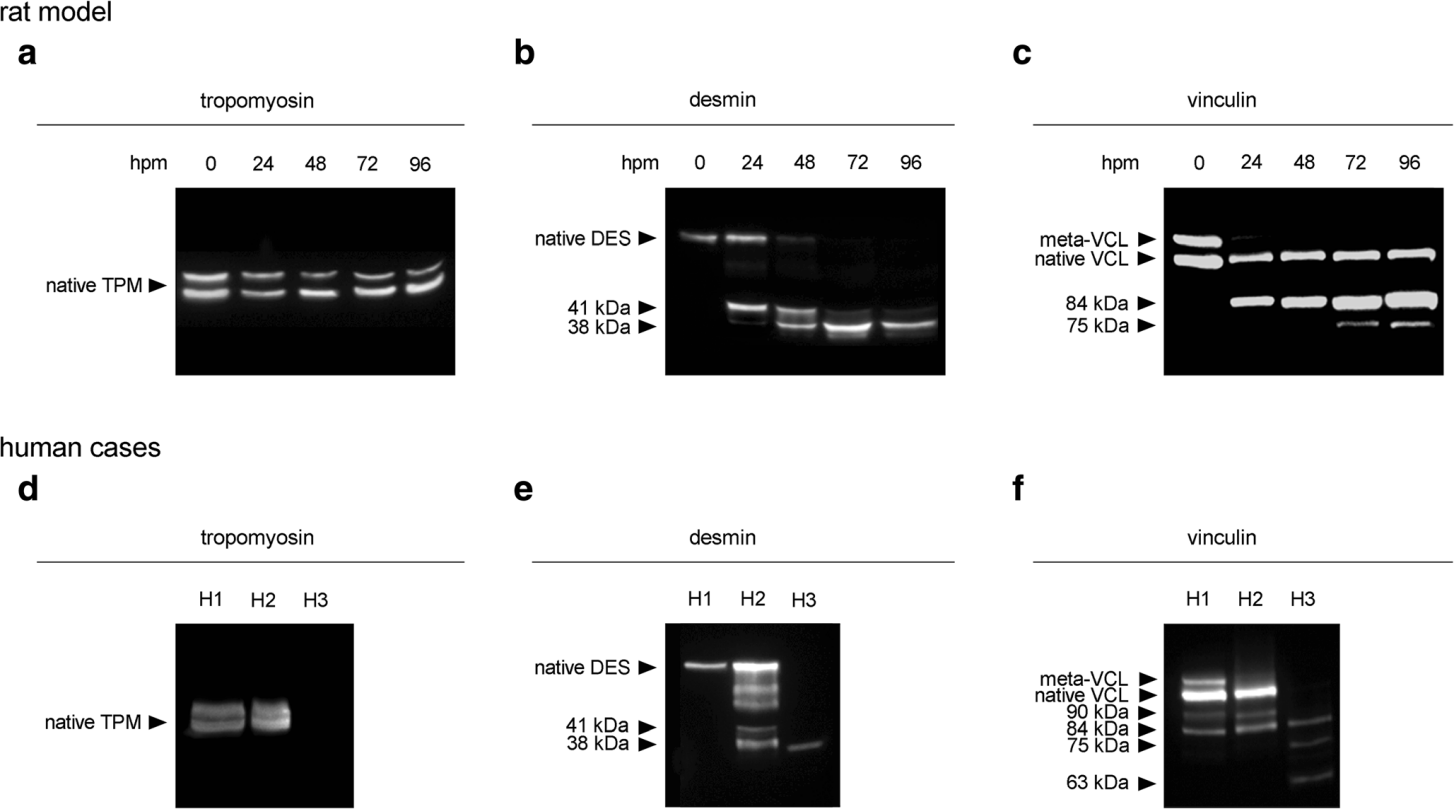
Western blot analysis of native standard proteins and degradation products in rats (*n* = 5) (**a**-**c**) and human cases (H1-H3) (**d**-**f**). Tropomyosin (TPM) (**a**) depicted no qualitative change of the characteristic double-band in any of the investigated samples and time points in rats. Desmin (DES) (**b**) and vinculin (VCL) (**c**) showed complete degradation of native bands (native DES and meta-VCL) as well as appearance of degradation products with increasing PMI (in hours postmortem, hpm). In human samples, TPM (**d**) depicted double bands in H1 and H2. In H3, no bands were present. Native DES bands (**e**) were found in H1 and H2, whereas in H3 the native band was lost. All human samples depicted characteristic DES degradation products of different molecular weights. Native VCL (**f**) was present in H1 and H2 and absent in H3. Meta-vinculin was only found in H1. Different sized characteristic degradation proteins were found in all human samples

**Fig. 3:**
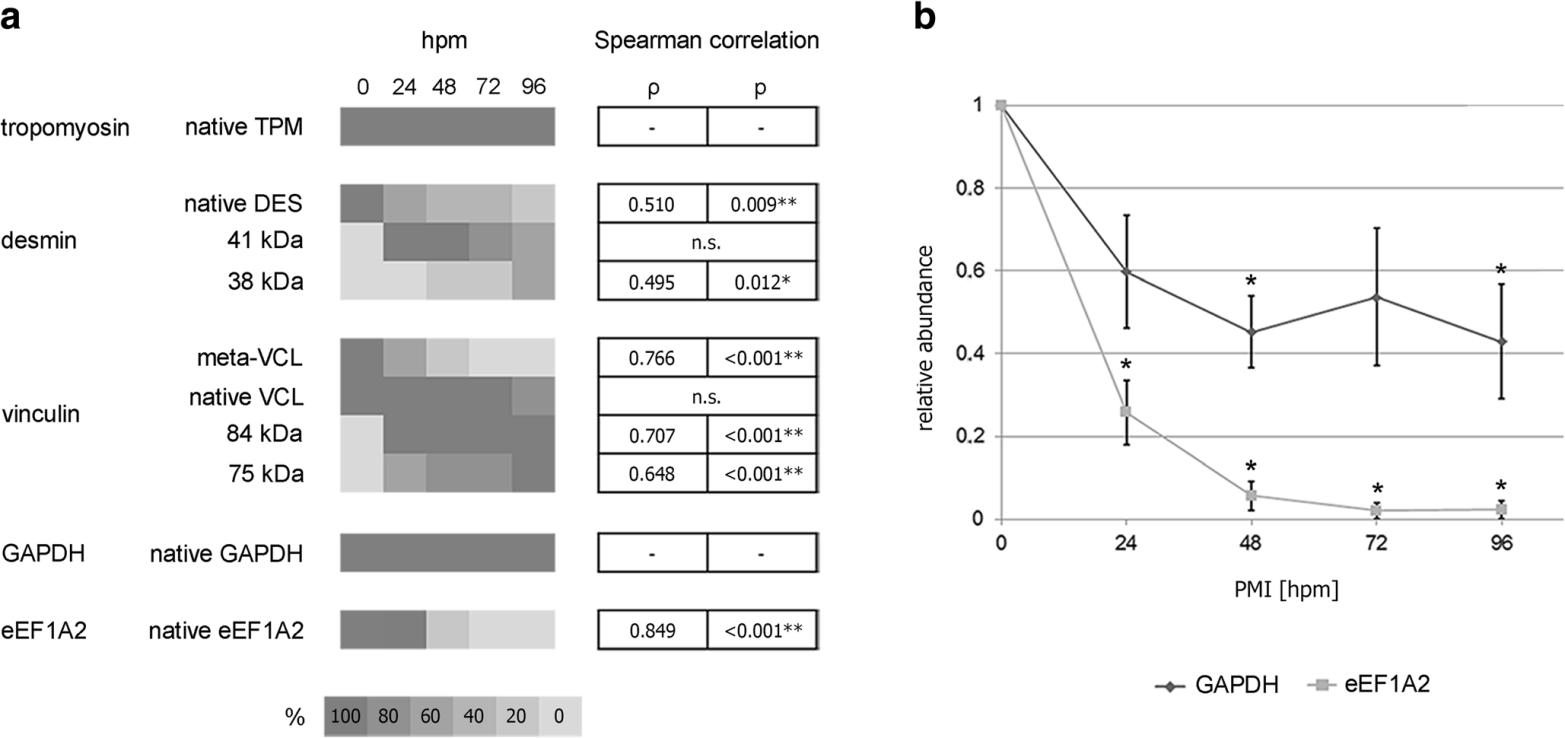
**a** Heat map depicting the frequency of band presence in all tested rat groups (*n* = 5 for each group). Tropomyosin (TPM) and GAPDH bands were detected in all, and native vinculin (VCL) in all but one analyzed sample and, thus, no correlation to the PMI was detected. However, native desmin (DES), meta-VCL, and native eEF1A2 depicted significant losses of the native band in correlation with the PMI. Additionally, significant correlations of the appearance of a 36 kDa desmin degradation product, and of 84 and 75 kDa vinculin degradation products, were detected. Asterisks mark significant (*) and highly significant (**) correlations, n.s. indicates not significant changes. **b** Relative abundance of GAPDH and eEF1A2 proteins over a PMI of 96 hpm in all tested rat groups (*n* = 5 for each group). Asterisks (*) indicate significant decreases of band intensity (relative abundance), as determined by Kruskal-Wallis test (*p* < 0.05). Data represented as mean ± SD

To validate postmortem degradation of GAPDH and eEF1A2 and to compare the degradation patterns to that of other well-established proteins on behalf of postmortem degradation (tropomyosin, desmin, vinculin), we ran standard Western blot analysis on a larger sample set, as described earlier [15, 27]. Tropomyosin western blots resulted in characteristic double bands in all animals and time points investigated (Fig. 2a). Desmin depicted a distinct single native band at approximately 50 kDa in all 0-hpm samples (Fig. 2b). In samples with PMIs between 24 and 72 hpm, the presence frequency decreased and at 96 hpm no native band was detectable in any of the samples. This represented a highly significant correlation with the time since death (*ρ* = 0.510, *p* = 0.009) (Fig. 3a). In addition to the gradual disappearance of the native band, all samples with a PMI of 24 hpm or more depicted characteristic degradation products. One band at approximately 41 kDa was present in all samples at 24 and 48 hpm, but gradually decreased in frequency in later time points. This transient appearance cannot be sensibly described using Spearman correlation. A second desmin degradation product with a molecular weight of approximately 38 kDa appeared initially in some of the 48-hpm samples and increased its occurrence frequency until 96 hpm, representing a significant correlation with the time since death (*ρ* = 0.495, *p* = 0.012) (Fig. 3a). Similarly, analysis of vinculin resulted in distinct native bands at approximately 117 and 135 kDa in all of the 0-hpm samples (Fig. 2c). These bands were recognized to represent the native form of vinculin (approximately 117 kDa) and the slightly larger splice variant meta-vinculin (approximately 135 kDa). Only few of the samples depicted a loss of the native vinculin band at 96 hpm. However, abundance of meta-vinculin bands significantly decreased, starting at 24 hpm and none of the samples collected from animals after 72 hpm depicted this band anymore. Spearman’s *ρ* for this protein band was very high and the PMI correlation highly significant (*ρ* = 0.766, *p* < 0.001) (Fig. 3a). Similar values were obtained for the appearance of two vinculin degradation products. While one degradation product at approximately 84 kDa was present in all samples with a PMI of 24 hpm and more (*ρ* = 0.707, *p* < 0.001), the second band at approximately 75 kDa gradually appeared in samples with PMIs of 24 to 72 hpm and was present in all animals at 96 hpm (*ρ* = 0.648, *p* < 0.001) (Fig. 3a).

**Fig. 4:**
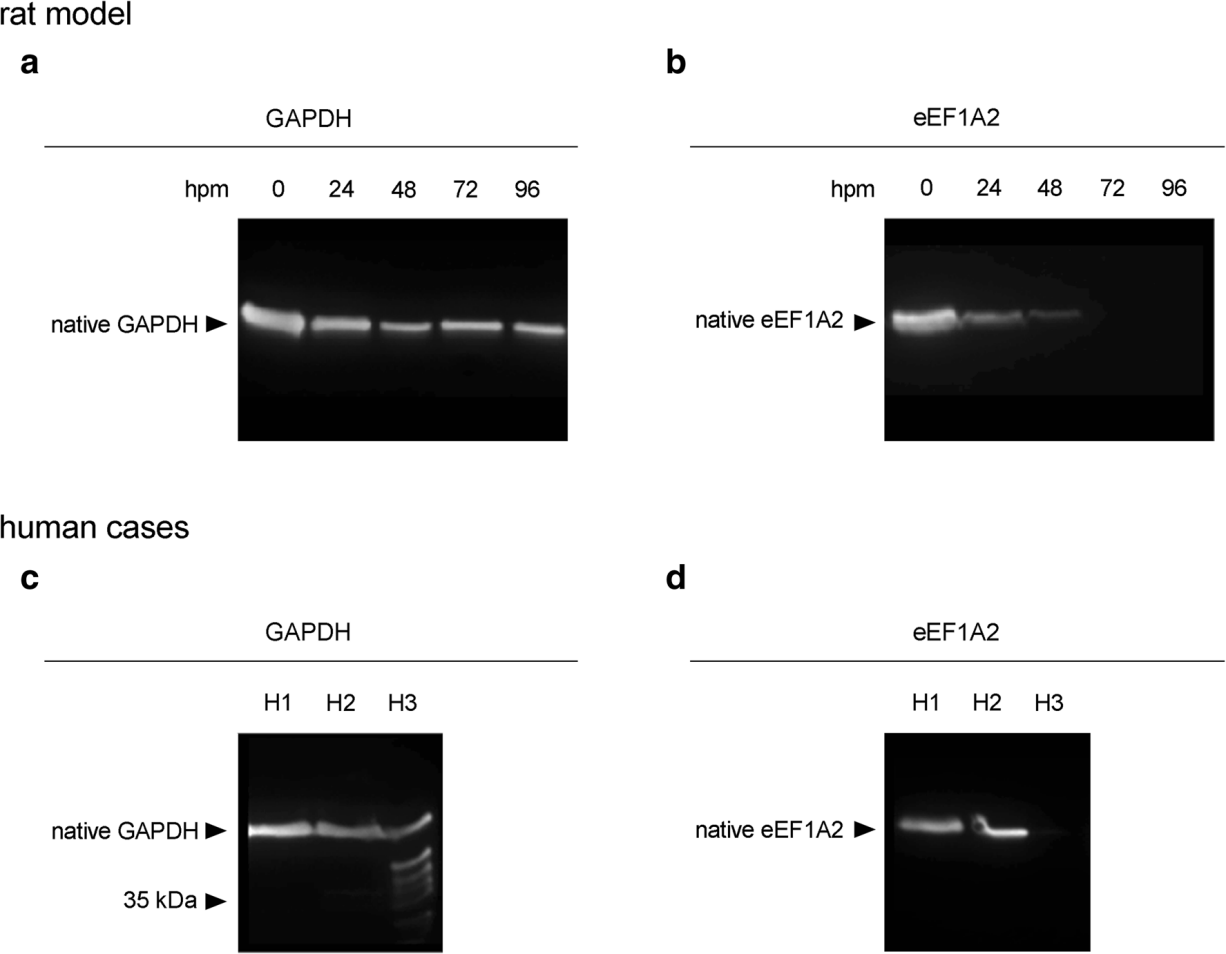
Western blot analysis of native proteins and degradation products of identified marker proteins GAPDH and eEF1A2 in rats (*n* = 5) (**a**, **b**) and human cases (H1-H3) (**c**, **d**).While GAPDH (**a**) depicted only a minor decrease of band intensity between 24 and 96 hpm, eEF1A2 in rats (**b**) showed a decrease below a 5% detection threshold (regarded loss of the band) in all rat 72- and 96-hpm samples. In human samples, there was no obvious difference detectable between H1 and H2 in GAPDH (**c**) and eEF1A2 (**d**). However, in H3, distinct GAPDH degradation products appeared (**c**) and no native eEF1A2 (**d**) was detected

Analysis of postmortem GAPDH band patterns resulted in distinct single bands at approximately 40 kDa (Fig. 4a). Bands with an intensity of > 5% of the native band were detected in all tested animals within the investigated time period of 96 hpm, and thus no correlation with the PMI could be calculated using these strict standard criteria of a significant qualitative change. However, there was a decrease of band intensity already at 24 hpm (mean 59.8% ± 2.9% of the native band) (Fig. 3b). This trend continued throughout the investigated period of 96 hpm and reached significance levels at 48 and 96 hpm respectively (*p* = 0.012 and *p* = 0.016). At 96 hpm, the mean intensity of the native GAPDH band was 42.9 ± 13.8% of the native band at 0 hpm.

eEF1A2 was detected as a distinct single band at approximately 50 kDa in all of the 0-hpm samples, and in significantly lower intensity in all 24-hpm samples (*p* < 0.001) (Fig. 3b, Fig. 4b). While in the 48-hpm samples, two of the five animals (40%) depicted bands above the threshold value of 5% of the native band (significantly decreased intensity, *p* < 0.001), no signal was detected in the other 48-hpm samples and in any of the 72- and 96-hpm samples, representing a highly significant correlation with the PMI (*p* < 0.001) with a Spearman *ρ* of 0.849 (Fig. 3a).

### Postmortem protein degradation in human cases

Similar to the animal model, we used the standard proteins tropomyosin, desmin, and vinculin to compare and validate postmortem degradation of GAPDH and eEF1A2 in human cases. Analyses of tropomyosin resulted in double bands in H1 and H2. In H3, tropomyosin bands were lacking (Fig. 2d). Western blot analysis of desmin showed a characteristic native band with a molecular weight of approximately 50 kDa in H1 and H2 (Fig. 2e). H2, in addition, depicted characteristic desmin degradation products of approximately 41 kDa and 38 kDa. In H3, only the 38 kDa band was present. Analysis of vinculin resulted in the native form of the protein in H1 and H2 (approximately 117 kDa) (Fig. 2f). In H1, additionally the higher molecular weight band was present (meta-vinculin, approximately 135 kDa). Both H1 and H2 samples also depicted protein bands at approximately 90 kDa and 84 kDa. Analysis of H3 revealed bands at approximately 84 kDa, 75 kDa, and 63 kDa.

To test the relevance of application in forensic cases, we analyzed the expression of GAPDH and eEF1A2 in three human cases representing different PMIs (Table 1). GAPDH protein patterns depicted distinct native bands in all three cases without major differences in intensity (Fig. 4c), confirming general applicability of this specific antiserum for human muscle tissue. In H3, several distinct degradation products of molecular weights between approximately 37 and 35 kDa were detected in addition to the native band. When analyzing postmortem eEF1A2, a distinct single band in H1 and H2 was detected (Fig. 4d), proving general applicability of this specific antiserum for forensic analysis. Notably, there was no obvious difference in band intensity detectable between both of these cases. However, in H3, no eEF1A2 band signal was detectable, suggesting similar processes in humans compared with the results from proteomic analysis and Western blot experiments in the animal model.

## Discussion

Protein-based analysis for time since death estimation has emerged as a promising tool in recent years, providing a, yet limited, spectrum of target proteins that are known to undergo predictable proteolysis in the early and mid-postmortem phase. Aiming to expand the insights into systemic postmortem protein alterations in skeletal muscle tissue, the present study demonstrates an efficient MS-based proteomics approach to characterize additional marker proteins for future use in PMI delimitation to extend the toolset for protein degradation–based time since death estimation method. By means of this target-based and unbiased system wide protein analysis, we were able to identify the new markers GAPDH and eEF1A2. By establishing a standardized rat degradation model, we generated a suitable model for testing the proteins under laboratory conditions and created the basis for testing the forensic applicability of the marker proteins in a pilot experiment using three autopsy cases. By performing SDS-PAGE and Western blot experiments, we proved the potential of both of these proteins to act as future markers for postmortem alterations in skeletal muscle and, thus, recommend this approach for the characterization of further reliable marker proteins applicable in practical forensic PMI delimitation.

GAPDH, one of the two newly discovered markers, is a widely used housekeeping protein, and antibodies against GAPDH are often used as a loading control in Western blot experiments because of its ubiquitously and constitutively expression in mammalian body cells [28, 29]. Antibodies against GAPDH are thus commonly available, and the protein is easily detectable by the Western blot technique. In contrast to other common housekeeping proteins, GAPDH is reported to be relatively stable in postmortem tissue of humans (brain tissue, 48 hpm) [30], which is in accordance with our qualitative evaluation of the results of GAPDH in H3 (PMI > 14 dpm). However, from the low number of human samples in this study, it cannot be concluded whether there is a statistical decrease of the native band in later postmortem stages, as it was the case in rat vastus lateralis muscle. The significant decomposition of GAPDH protein in rats over a PMI of 96 hpm is confirmed by the proteomic analysis and by a study reporting postmortem GAPDH decrease in rat psoas muscle [17] over a similar period of time. Despite the trend to decrease, the presence of the native band of GAPDH band at a PMI of 96 hpm and the occurring degradation products at a larger PMI in the human sample H3 indicate possible applicability as a marker in later postmortem stages. Especially, the degradation products depict a qualitative change of band pattern that, in other proteins, has been shown to be able to significantly contribute to estimate the time since death [27]. Additional experiments in wider time frames and a large sample size are required to determine whether there is a significant loss of the native band at later postmortem stages and whether the degradation products can be reproduced in human samples and may also appear in rat muscles in later stages. It has to be further evaluated whether their appearance is influenced by individual or environmental factors.

In contrast to the steadily decreasing character of GAPDH, the (early) loss of the translation factor eEF1A2 was found to be in strong correlation with the PMI, demonstrating the importance of this marker. Although eEF1A is found in high concentrations within body cells (3% of the total cellular protein) [31, 32], this is, to our knowledge, the first study on postmortem eEF1A2 decomposition. Proteomic investigations resulted in a successive decrease in the rat and mouse proteome, which was confirmed in Western blot experiments. Additionally, band intensities decreased below a 5% threshold from 48 hpm onwards and were, thus, considered a qualitative change (loss of a band). In contrast, in the human cases, we detected no obvious difference in eEF1A2 band intensity between H1 and H2, underlining the requirement of strict criteria for postmortem changes of protein band patterns for the use in forensic casework. Therefore, we generally suggest that qualitative changes (loss of a band, or appearance of a degradation product) should be preferred over quantitative measurements (e.g., merely a decrease in band intensities). However, additional experiments are required to characterize the precise timeframe in which the eEF1A2 band loss occurs in humans, and whether or how it is influenced by certain individual or environmental factors. Additional caution has to be taken when analyzing samples from cancer patients, as eEF1A2 can be overexpressed in such cases [33]. Additional research has to evaluate whether this might be an exclusion criteria for the use of this marker.

When comparing the results to already established markers (tropomyosin, desmin, and vinculin), it was detected that the loss of the native eEF1A2 band in rats depicted the strongest correlation values with the PMI. This resulting correlation could though be influenced by the specific temporal degradation characteristics of the native eEF1A2 band (initial band loss at 48 hpm, complete band loss at 72 hpm) and the selected statistic test in the present study. Comparable alterations, such as the loss of meta-vinculin, and the occurrence of the 84 kDa vinculin degradation product are, nevertheless, equally important markers for time since death estimation although resulting in lower correlation values. In the case of the 84 kDa vinculin band within the rat model, which was negative (−, no measureable band) in all 0-hpm samples and positive (+, measurable band) in all samples with larger PMI, lower correlation values resulted from a less balanced overall ± ratio. Still, in accordance with previous studies [16], all results of the animal model were found to significantly correlate with the PMI.

Changes, such as the transient appearance of the 41 kDa band in the cytoskeletal protein desmin, could not be described using Spearman correlations. However, if certain timeframes can be excluded in future application, such transiently appearing protein bands can be of potential interest. The transient character of the 41 kDa desmin degradation product in this experimental series was in accordance with earlier studies [16, 34]. Together with the loss of the native band and the appearance of the 34 kDa band (both significant correlations with the PMI), it could clearly be confirmed that desmin is a reliable marker for early phase time since death estimation. Tropomyosin results were as well in accordance with previous studies reporting postmortem stability of this protein until 240 hpm [15]. Although a real time passage (analysis of multiple sampling time points of one individual) is lacking for humans, selected cases with different PMI/ADD clearly demonstrated predictable degradation pattern of all tested standard proteins which are comparable to those in the rat model and also largely in accordance with previous studies on human muscle samples [35]. Notably, there were no tropomyosin bands detected in H3. Whether this represents a characteristic degradation pattern for larger PMIs remains to be determined.

It can be concluded that LC-MS/MS analysis of postmortem skeletal muscle samples is a valuable method for the identification of biomarkers for forensic time since death estimation by analysis of protein degradation. With the cost-effective method we presented here, we were able to characterize promising marker candidates for further research in an appropriate timeframe. By running additional routine experiments, we confirmed significant correlations of the new identified marker proteins GAPDH and eEF1A2 with the PMI in an animal model. Although the animal models used in this study are distinct to humans, we were able to demonstrate similar degradation pattern of these proteins in humans. With this, we prove their relevance in future forensic application and generally strengthen the applicability of animal models for basic research on PMI estimation as indicated by a previous work [34]. For future characterization of additional markers using proteomics, we recommend to consider additional experiments focusing as well on different species, extended time frames, or varying environmental conditions. Especially, investigations on the human postmortem proteome could, in future experiments, crucially contribute to the characterization of important additional biomarkers.

## Electronic supplementary material

ESM 1 (XLSX 11 kb)

ESM 2 (DOCX 19 kb)
