# Supplementary material for: Postmortem proteomics to discover biomarkers for forensic PMI estimation
Source: Int J Legal Med. 2019 Mar 12;133(3):899–908. doi: 10.1007/s00414-019-02011-6 (PMC6469664; doi:10.1007/s00414-019-02011-6)
Supplement: Supplementary file 2 — (DOCX 19 kb) [file 414_2019_2011_MOESM2_ESM.docx]

**LC-MS/MS analysis**

Peptides were trapped at 75 μm × 2 cm C18 precolumn (for details of products and specific solutions see Online Resource 1) before being separated on an analytical C18 column at a flow rate of 300 nL/min. The mobile phases A and B were composed of 0 and 80% acetonitrile containing 0.1% formic acid, respectively. The LC gradient began with 2% B and was ramped to 6% B for 1 min, to 10% B for 16 min, to 50% B for 74 min, to 100% B for 1 min, and remained at 100% B over 8 min. Finally, it was ramped to 2% B for another 5 min. The column was re-equilibrated with 2% B for 15 min before the next run. The voltage applied to produce an electrospray was 1900 V. During the chromatographic separation the Orbitrap Fusion Lumos was operated in data-dependent mode, automatically switching between MS1 and MS2. The MS data were acquired using the following parameters: Full scan MS1 spectral (400-1600 m/z) were acquired in the Orbitrap for a maximum ion injection time of 100 ms at a resolution of 120,000 and an automatic gain control (AGC) target value of 4.0e5. MS2 spectra were acquired in the Orbitrap mass analyzer at resolution of 30,000 with high energy collision dissociation (HCD) of 27% normalized collision energy and AGC target value of 5.0e4 with maximum ion injection time of 54ms. Previously fragmented ions were excluded for 12 sec.

**Data processing**

The proteins identified in the mass spectrometry data were analyzed and quantified using MaxQuant Version 1.2.2.5 [1] as described previously [2]. Briefly, enzyme specificity was set to full trypsin digestion, allowing up to 2 missed cleavages. Carbamidomethylation of cysteine was set as a fixed modification; oxidation of methionine and N-terminal protein acetylation were included as variable modifications. First search ppm for the peptide mass tolerance was set to 20 ppm, and fragment ion tolerance was set to 0.05 Da. Protein and peptide false discovery rates were set to 0.05, and minimum peptide length was set to 6 amino acids. MS/MS data were searched against the Swiss-Prot human database combined with common contaminants and concatenated with the reversed versions of all sequences. The resulting “ProteinGroups” files were used for further analysis. First, LFQ-intensities of each protein were normalized (log2 scale) based on total intensity (TI) employing Normalizer tool [3]. Second, the normalized protein list was clustered using an unsupervised hierarchical method with a Euclidian distance function (R script). Third, we sought to identify proteins whose intensities were decreased as hours post mortem (hpm) increase. For each protein, we obtained protein intensity changes from the baseline intensity by subtracting the baseline intensity (0 hpm) from 0 hpm-96 hpm for each protein (log_2_ 96 hpm – log_2_ 0 hpm) = log_2_ (96 hpm/0 hpm). Then, we selected proteins whose intensities were monotonically decreased (i.e. 0 hpm intensity ≥ 24hpm intensity ≥ 48hpm intensity ≥ 72 hpm intensity ≥ 96 hpm intensity) using a MATLAB script.

1. Cox J, Mann M (2008) MaxQuant enables high peptide identification rates, individualized p.p.b.-range mass accuracies and proteome-wide protein quantification. Nat Biotechnol 26:1367–1372. https://doi.org/10.1038/nbt.1511

2. Kim J-Y, Welsh EA, Fang B, et al (2016) Phosphoproteomics Reveals MAPK Inhibitors Enhance MET- and EGFR-Driven AKT Signaling in KRAS-Mutant Lung Cancer. Mol Cancer Res MCR 14:1019–1029. https://doi.org/10.1158/1541-7786.MCR-15-0506

3. Chawade A, Alexandersson E, Levander F (2014) Normalyzer: a tool for rapid evaluation of normalization methods for omics data sets. J Proteome Res 13:3114–3120. https://doi.org/10.1021/pr401264n
